# Small-Molecule Modulation of PPARs for the Treatment of Prevalent Vascular Retinal Diseases

**DOI:** 10.3390/ijms21239251

**Published:** 2020-12-04

**Authors:** Xiaozheng Dou, Adam S. Duerfeldt

**Affiliations:** 1 Department of Chemistry & Biochemistry, University of Notre Dame, Notre Dame, IN 46656, USA; xdou@nd.edu; 2 Department of Chemistry & Biochemistry, University of Oklahoma, Norman, OK 73019, USA

**Keywords:** diabetic retinopathy, age-related macular degeneration, retinopathy of prematurity, peroxisome proliferator-activated receptor, drug discovery

## Abstract

Vascular-related retinal diseases dramatically impact quality of life and create a substantial burden on the healthcare system. Age-related macular degeneration, diabetic retinopathy, and retinopathy of prematurity are leading causes of irreversible blindness. In recent years, the scientific community has made great progress in understanding the pathology of these diseases and recent discoveries have identified promising new treatment strategies. Specifically, compelling biochemical and clinical evidence is arising that small-molecule modulation of peroxisome proliferator-activated receptors (PPARs) represents a promising approach to simultaneously address many of the pathological drivers of these vascular-related retinal diseases. This has excited academic and pharmaceutical researchers towards developing new and potent PPAR ligands. This review highlights recent developments in PPAR ligand discovery and discusses the downstream effects of targeting PPARs as a therapeutic approach to treating retinal vascular diseases.

## 1. Introduction 

Vision provides ~80% of the information acquired from the environment and is arguably the most important sensory function for maintaining a high quality of life [1]. Progressive irreversible blindness or significant visual impairment results in drastic changes to lifestyle that can lead to additional hardships (e.g., financial, familial, logistical), especially in the working-age population [2]. Thus, although visual impairment produces a substantial burden on the healthcare system (USD 5.8 billion was reimbursed for ophthalmology in 2013 in the U.S.), the overall impact is immeasurable [3]. Two diseases that contribute the most to pathological blindness are diabetic retinopathy (DR) and age-related macular degeneration (AMD), both of which result from atypical vasculature and retinal damage [4]. Additionally, retinopathy of prematurity (ROP), a condition driven by similar pathological features, is becoming increasingly common in the neonatal population [5]. Thus, vascular-related retinal diseases affect patient populations at both ends of the age spectrum and novel strategies to prevent, treat, and reverse diseases are needed. The goal of this review is to summarize recent evidence that small-molecule modulation of peroxisome proliferator-activated receptors (PPARs) represents a promising approach worthy of continued pursuit for these vascular-related retinal diseases. 

## 2. Age-Related Macular Degeneration (AMD)

AMD is caused by deterioration of photoreceptor cells in the macula due to abnormalities within the retinal pigment epithelium (RPE), leading to central vision loss [6,7,8]. Currently, AMD is estimated to affect ~196 million people worldwide [9]. Due to an aging population, this number is expected to climb to ~288 million by 2040 [6,10]. Genetic and/or environmental factors are both thought to play significant roles in AMD [11]. During the earliest stages of AMD, insoluble extracellular aggregates (i.e., drusen) form in the retina but no signs of RPE abnormalities or vision loss are apparent [12,13]. In the intermediate stages of AMD, drusen enlarge, resulting in RPE abnormalities and increasing the risk for progression to late-stage AMD [12,13]. Patients with late-stage AMD exhibit one of two forms, namely geographic atrophy (i.e., dry AMD), or neovascular (i.e., wet AMD), either of which results in progressive vision loss [14,15]. In dry AMD, degeneration of RPE cells leads to the destruction of light-sensing retinal photoreceptors, resulting in gradual vision loss. In contrast, acute vison loss resulting from wet AMD arises from the abnormal growth of blood vessels in choroids, termed choroidal neovascularization (CNV) [6,7,16]. Overall, the pathobiology of AMD is multi-faceted and involves: oxidative damage, lipofuscin accumulation, impaired activity or function of the RPE, increased apoptosis, abnormal immune system activation, senescent loss of homeostatic control, and/or abnormalities in Bruch’s membrane [7,10]. 

In 2006, the US Food and Drug Administration (FDA) approved ranibizumab (Lucentis, Genentech), a vascular endothelial growth factor A (VEGF-A) antibody, providing a breakthrough treatment for AMD. VEGF is a signaling protein produced by cells that stimulates the formation of blood vessels and is comprised of five members: VEGF-A, VEGF-B, VEGF-C, VEGF-D, and placenta growth factor (PGF) [17]. Specifically, VEGF-A has been implicated in CNV and in the increased vascular permeability that results in eventual loss of vision, and thus is recognized as a central contributor to the pathology of wet AMD [18,19,20,21,22]. FDA-approved anti-VEGF agents now include pegaptanib sodium (Macugen, Eyetech/Pfizer), ranibizumab (Lucentis, Genentech/Roche), aflibercept (Eylea, Regeneron), and bevacizumab (Avastin, Genentech) (Table 1). It is interesting to note that bevacizumab is less expensive (USD 50/dose) than ranibizumab (USD 2000/dose) and exhibits a similar improvement in visual acuity (7.8 compared to ranibizumab at 8.8), but is not approved for AMD [23]. 

Although anti-VEGF approaches have drastically improved the quality of life for many patients with wet AMD, they fail to address inflammation, apoptosis, and oxidative damage in the retinal disorders [17]. Moreover, patients with dry AMD remain refractory to anti-VEGF-centered treatments [10]. In fact, there is no treatment available to prevent or reverse the progression of dry AMD. Moreover, long-term anti-VEGF treatment has now revealed various complications, such as endophthalmitis, retinal and retinal pigment epithelial detachment, retinal pigment epithelial tears, anterior chamber inflammation, increased intraocular pressure, and intraocular hemorrhage [24].

## 3. Diabetic Retinopathy (DR)

DR is one of the most common complications of diabetes and the primary cause for vision impairment in the working-age population worldwide. The number of patients globally with DR is estimated to exceed 160 million people [25,26,27]. Considering the growing prevalence of diabetes, DR will continue to produce a large burden on healthcare until new countermeasures are developed. To this point, the World Health Organization (WHO) has called for global action to halt the increase in diabetes by 2025 and improve care for complications arising from diabetes [28].

Diabetic macular edema (DME), caused by retinal vascular leakage and neovascularization, is the major pathological feature responsible for DR-induced vision loss [29]. DME leads to retinal ischemia and increased levels of VEGF, which results in the development of aberrant neovascularization. The severity of DR is classified into two categories: non-proliferative diabetic retinopathy (NPDR) and proliferative diabetic retinopathy (PDR). NPDR comprises the early stage of DR and is characterized by micro-aneurysms, retinal hemorrhages, and exudates. Abnormal retinal blood vessel formation synergizes with an increase in intraocular VEGF levels, eventually leading to PDR, characterized by aberrant retinal neovascularization. If neovascularization is left untreated, vitreous hemorrhage and retinal detachment can occur, eventually producing extensive retinal damage and blindness. Accumulating evidence suggests that DR exhibits characteristics of chronic inflammation, as multiple pro-inflammatory factors such as tumor necrosis factor-α (TNF-α), [30,31] intercellular cell adhesion molecule-1 (ICAM-1), [32] and VEGF [33] are overexpressed in the diabetic retina [29]. Retinal inflammation plays a causative role in an impaired vascular endothelium, pericyte loss, vascular leakage, and later retinal neovascularization [29,34,35].

Despite standard treatment options, including laser photocoagulation, glucose-lowering treatments, and intravitreal injection of corticosteroids and anti-VEGF antibodies, the ability to address the complex nature of DR remains a challenge [34]. In fact, > 40% of the DR patient population fails to respond to the gold-standard anti-VEGF treatment [36]. As mentioned in the context of AMD, evidence is mounting that long-term anti-VEGF therapy can lead to cataracts, infection, vitreous hemorrhage, fibrosis, and even retinal detachment [37]. Additional studies that define potential long-term complications (i.e., hypertension, proteinuria, ischemic cardiovascular disease) induced by anti-VEGF agents due to systemic exposure are still needed [38]. Another common treatment, laser photocoagulation, suffers from its destructive nature that commonly leads to the exacerbation or development of macular edema, and loss of peripheral and/or night vision [39]. 

## 4. Retinopathy of Prematurity (ROP)

Due to modern medical advances, we are now able to save exceedingly premature neonates. Premature infants are at a higher than average risk for developing retinopathy of prematurity (ROP), a condition resulting from eye vascular abnormalities that can lead to blindness [40]. The National Eye Institute estimates that nearly 16,000 of the 3.9 million infants born in the U.S. suffer from some degree of ROP each year [41] Of the ROP diagnosed infants, ~10% will require medical treatment and ~4% will become legally blind due to ROP-related issues, [41] with low- and middle-income countries exhibiting a blindness prevalence of ~40% [42].

Scarring and retinal detachment observed in ROP are caused by disorganized growth of retinal blood vessels during premature development. The first phase arises from vaso-obliteration of the developing retinal capillaries due to decreased levels of cytoprotective factors. This leads to hypoxic vasoproliferation in the second phase, wherein the hypoxic retina overproduces hormones (e.g., VEGF), resulting in the growth of anarchic vessel formation at the immature nonperfused area of the retina. Eventually, abnormal neovasculature accumulates in the retina, leading to final invasion into the vitreous, which causes blindness [40,43]. 

The current gold-standard treatments for ROP are cryotherapy and laser photocoagulation. The two approaches destroy the portion of the avascular retina that is the source of growth factors, which promote neovascularization [44]. This results in irreversible damage to the peripheral retina, significantly reducing vision. Additionally, laser photocoagulation has been shown to be a major contributor to the development of corneal edema, myopia, intraocular hemorrhage, and cataract formation [45,46].

As expected, anti-VEGF strategies have recently been pursued as a preventative and less destructive therapy for ROP [46,47]. While anti-VEGF injections reduce the risk of recurrence in infants with zone I ROP, an increase in recurrence for infants with zone II ROP has been observed [48]. Moreover, when anti-VEGF agents were used as a monotherapy, neither bevacizumab nor ranibizumab reduced the risk of retinal detachment, mortality before discharge, corneal opacity requiring corneal transplant, or lens opacity requiring cataract removal [48]. Furthermore, recent evidence suggests that anti-VEGF treatments exhibit not only ocular issues but also result in systemic complications in premature infants [49]. Therefore, in addition to potential ethical issues pertaining to this treatment method in infants, the safety and efficacy of anti-VEGF agents raise significant concerns.

## 5. Retinal Diseases: State of Treatment

Despite a number of treatment options, the ability to address the complex nature of retinal diseases such as DR, AMD, and ROP remains a significant challenge [34]. Frontline approaches require frequent injections, are destructive, demand specialized facilities, suffer from limited response rates, and/or produce significant financial burdens on the healthcare system. Not unexpected, since VEGF-A has been reported to have neurotrophic roles in the retina, VEGF inhibition is known to exhibit dose-dependent toxicity against multiple retinal cell types in rodents [50,51]. Thus, a critical need exists to develop new treatments. 

Looking to the future of retinal disease treatment, emerging paradigms should diverge from the conventional approaches of simply preventing a singular etiological feature (e.g., disrupting angiogenesis through VEGF neutralization). Therapeutic approaches that synergistically protect the retina from inflammation, cell death, leakage, and angiogenesis are likely to dramatically improve disease outcomes, and thus quality of life. Unlike current options, these new approaches should be non-invasive (to the eye), safe, readily available, affordable, and capable of being administered without specialized facilities. New therapies that are either superior to or synergistic with current approaches will allow for the treatment of a wider population demographic by addressing those not suitable for current approaches and will be of great value to patients. 

## 6. A Case for Targeting PPARs

**Figure 1:**
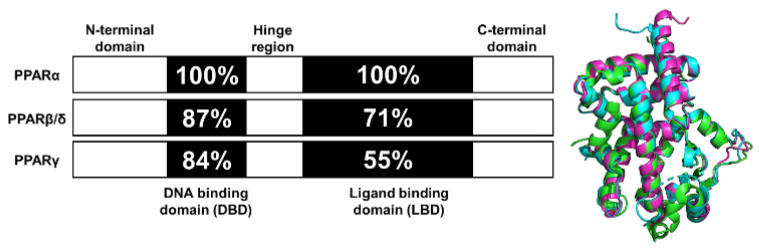
Functional domains and secondary structures of PPAR isoforms. Numbers denote residue identity in percent compared to PPARα. Right is the overlap of the ligand-binding domain (LBD) for all PPAR isoforms. PPARα-LBD (green, PDBID: 2P54), PPARβ/δ-LBD (cyan, PDBID: 3TKM), and PPARγ-LBD (magenta, PDBID: 2VV0).

Peroxisome proliferator-activated receptors (PPARs) are a group of ligand-dependent nuclear transcription factors that play essential roles in regulating the energy balance and metabolic processes. As such, PPARs have received a significant amount of attention as drug targets for diseases ranging from dyslipidemia to Alzheimer’s [52,53]. Three PPAR subtypes exist and are the products of distinct genes, commonly identified as PPARα (NR1C1), PPARβ/δ (NR1C2), and PPARγ (NR1C3). All PPARs exhibit a prototypical domain architecture including (i) an N-terminal region, (ii) a highly conserved DNA-binding domain (DBD), (iii) a flexible hinge region, (iv) a ligand-binding domain (LBD), and (v) a C-terminal region (Figure 1). To regulate gene expression, PPARs often form a heterodimeric complex with retinoid X receptor (RXR) [53,54]. The heterodimer is activated by binding of a ligand to PPAR and/or RXR. Activation results in corepressor dissociation and binding of the heterodimer to the peroxisome proliferator response elements (PPREs) on the promoter domain of the target genes, subsequently leading to gene transcription [53]. Due to different expression patterns, tissue distribution, and pharmacological profiles, each PPAR subtype regulates different metabolic pathways.

Over the last decade, accumulating evidence has indicated that small-molecule PPAR modulation can produce anti-angiogenesis, anti-fibrosis, anti-inflammation, and anti-oxidative effects in various organs. As mentioned earlier, the pathological mechanisms of major blinding diseases, such as ADM, DR, and ROP, often involve neovascularization, inflammation, and oxidative stress-mediated cell death. Thus, researchers have postulated that PPAR modulation represents a promising strategy to address these vision-threatening diseases in diverse patient populations through simultaneous regulation of many etiological features.

## 7. PPARβ/δ

PPARβ/δ is the least understood isoform in the PPAR family and is ubiquitously expressed. While historically referred to in the literature as both PPARβ and PPARδ (PPARβ/δ), we will refer to this isoform as PPARδ for clarity. This isoform regulates fatty acid catabolism, energy metabolism, and reverse cholesterol transport [55]. Activation of PPARδ was initially thought to be a viable therapeutic strategy for dyslipidemia, diabetes, and cardiovascular disease, but the beneficial effects in primate models could not be reproduced [56]. Recently, a growing body of evidence suggests that PPARδ is also involved in angiogenesis, inflammation, lipid metabolism, and extracellular matrix remodeling, which are central to the pathogenesis of retinal diseases such as AMD [57,58,59]. Inspired by these results, more studies have been carried out to determine the effects of PPARδ modulation in ophthalmology.

Work by Malek and colleagues demonstrates that PPARδ is a key contributor to the RPE and choroidal endothelial cell biology, two cell types compromised in AMD development and progression [57]. Knockdown of PPARδ results in an upregulation of extracellular matrix gene expression in primary RPE cells but a concomitant reduction in choroidal endothelial cells [57]. In both cell types, however, a downregulation of factors involved with angiogenesis, including VEGF-A, was observed following PPARδ knockdown, confirming that PPARδ is a driver of neovascular lesions [57]. This result was confirmed in vivo with aged *Pparδ^-/-^* mice. However, these *Pparδ^-/-^* mice were found to exhibit several phenotypic features of dry AMD including hypo- and hyper-pigmentation, loss of basal infoldings, thickened Bruch’s membrane, and a higher frequency of abnormal sub-RPE deposits [57]. The Wang group also reported that PPARδ plays a critical role in retinal blood vessel remodeling and pathological angiogenesis in mice [60]. Results from these studies demonstrate cell-specific effects arising from PPARδ inhibition, an observation that may be due to the differential expression of the receptors themselves or related regulatory factors (e.g., coactivators or co-repressors).

Malek and co-workers also assessed the effects of pharmacological modulation of PPARδ on choroidal neovascularization and lipid accumulation [57]. Inhibition of PPARδ was shown to decrease neovascular lesion formation and angiogenic factors and downregulate expression of extracellular matrix components, while agonism of PPARδ decreased lipid accumulation [57].

**Figure 2:**
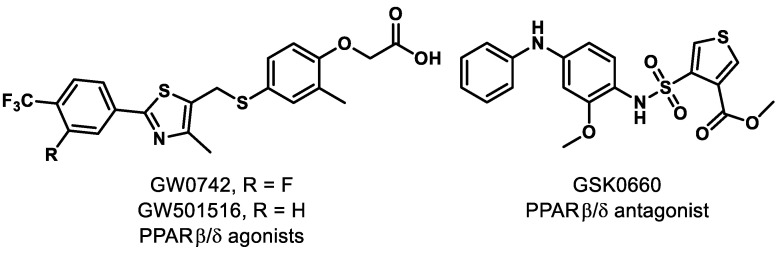
Representative PPARβ/δ modulators.

Separate investigations, however, have revealed that pharmacological PPARδ agonism aggravates angiogenic cell behaviors and oxygen-induced retinopathy (OIR). In fact, administration of PPARδ agonists GW0742 and GW501516 (Figure 2) significantly increased the level of angiopoietin-like-4 (angptl4) mRNA, which is known to increase tubulogenesis in human retinal microvascular endothelial cells (HRMECs) and OIR rats [61]. A similar result was reported in recent work, demonstrating that while PPARδ activation provides anti-inflammatory effects, it promotes neovascularization of alkali-injured eyes in a rat model [62].

On the other hand, pharmacological antagonism of PPARδ by GSK0660 (Figure 2) was reported to decrease the level of angptl4 mRNA and provide a concomitant reduction in proliferation and tubulogenesis in HRMECs and in preretinal neovascularization in OIR rats [61]. Penn and colleagues provided further evidence that PPARδ antagonism exhibits promise, as they observed that administration of GSK0660 decreased phosphorylation of extracellular signal-regulated protein kinases and expression of VEGF in HRMECs, and reduced retinal vascular permeability and retinal VEGF levels in a mouse model [63]. With these promising results, studies were conducted on the mechanism of vascular inflammation and PPARδ antagonism. It was concluded that GSK0660 prevents upregulation of TNFα-induced transcription, such as chemokine ligand 8 (CCL8), chemokine ligand 17 (CCL17), and C-X-C motif chemokine 10 (CXCL10), which inhibits leukocyte recruitment in HRMECs [64]. Although the evidence clearly suggests that the ubiquitously expressed PPARδ is a significant component in the initiation and progression of retinal diseases, the functional studies of PPARδ are still in their infancy and the ability to achieve tissue specificity of pharmacological modulators presents a challenge. The evidence for PPARδ antagonism as a novel therapeutic approach for retinal hyperpermeability is compelling.

## 8. PPARγ

PPARγ is arguably the most widely investigated PPAR subtype. It is expressed predominantly in adipose tissue, kidney, stomach, heart, liver, spleen, and brain [53]. The primary functions of PPARγ are to regulate energy storage and utilization, inflammatory and immunological responses, and adipocyte differentiation [53,65]. Molecular implications of PPARγ in retinal diseases have been reported thoroughly in several communications over the last decade [65,66,67,68]. Activation of PPARγ provides a neuroprotective effect and inhibits microvascular abnormalities in DR [67]. Moreover, research clearly demonstrates that PPARγ activation inhibits CNV, attenuates retinal and choroidal angiogenesis, and renews photoreceptor processes corrupted by oxidants in AMD [65]. Subsequent studies show that upregulation of PPARγ induces anti-fibrogenic effects in AMD models [69]. Given the downstream effects of PPARγ agonism and/or upregulation, the reasons for continued investigation into PPARγ and its therapeutic potential are compelling. It is worth noting that while PPARγ expression has been detected in human fetal RPE cells, human retinal samples (age unspecified), and cultured RPE and ARPE19 cells, expression was not detected in RPE cells isolated from fresh adult donors. Differences in expression levels could be due to a number of factors (e.g., age differences, population sample heterogeneity), but this observation highlights the importance of system compatibility and is likely to make data set comparisons difficult [70]. 

**Figure 3:**
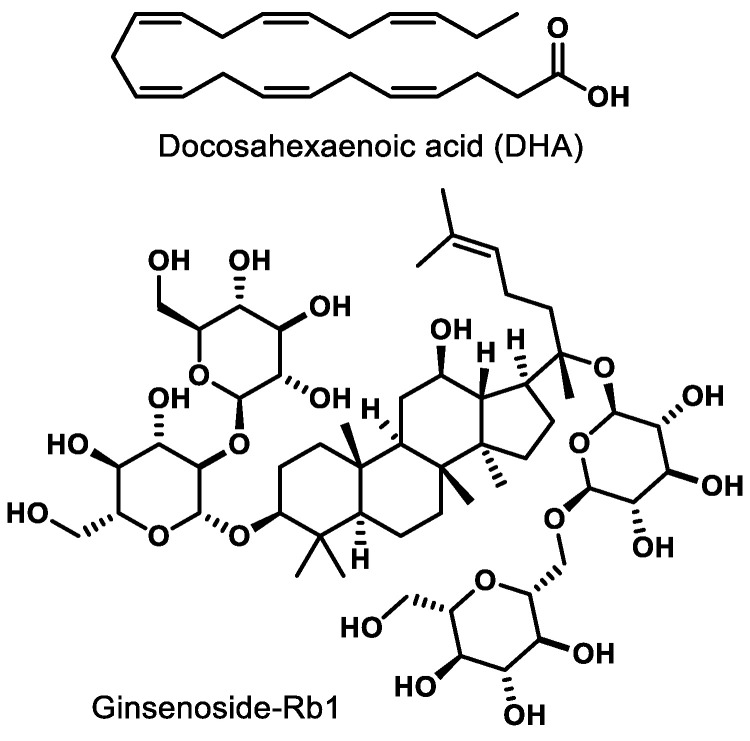
Representative natural product PPARγ ligands.

Docosahexaenoic acid (DHA, Figure 3), a naturally occurring omega-3-fatty acid, is an agonist of PPARγ. In newborn Sprague-Dawley rats, agonism of PPARγ by DHA decreases nuclear factor-kappa B (NF-κB) activity, leading to inhibition of advanced glycation products (AGE) known to induce microglia activation in retinal cells [71]. Ginsenoside-Rb1 (Rb1, Figure 3), the most abundant ginsenoside isolated from *Panax ginseng*, elicits an anti-angiogenic effect in human umbilical vein endothelial cells (HUVECs). This effect is believed to be due to the ability of Rb1 to increase pigment epithelial-derived factor (PEDF) expression and reduce miR-33 through a PPARγ-dependent pathway [72]. These results arising from two different natural products demonstrate that either direct (e.g., DHA) or indirect (Rb-1) modulation of PPARγ has potential to mitigate pathological features exhibited by common retinal diseases.

**Figure 4:**
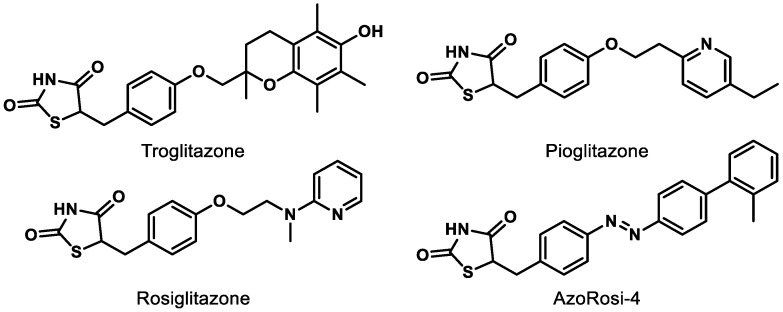
Representative synthetic PPARγ agonists belonging to the thiazolidinedione (TZD) chemotype.

Although natural products and a long list of synthetic rationally designed PPARγ ligands have been assessed at the cellular and pre-clinical levels, the thiazolidinediones (TZDs) remain the most well-studied chemotype. This class of synthetic PPARγ agonists includes rosiglitazone and troglitazone (Figure 4) and has been assessed for efficacy against a number of metabolic conditions in humans since the late 1990s [73]. While TZDs exhibit efficacy in retinal disease models, [65,73] they are known to exhibit numerous adverse side effects (e.g., increased macular edema, bone fracture, congestive heart failure) and thus face high levels of scrutiny from the FDA [65,74]. Therefore, selective PPARγ agonists and dual PPAR agonists that include PPARγ agonism are likely to continue to face a precarious journey for drug development unless these effects can be determined to be chemotype-specific. Photoswitchable PPARγ selective agonists (e.g., AzoRosi-4, Figure 4), elegantly designed by Trauner and colleagues, represent an exciting new chemotype to explore, especially because the light-driven behavior of this class is engineered to potentially provide eye-specific activity [75]. 

## 9. PPARα

PPARα is the most studied isoform with respect to retinal diseases, with published results tracking back to 1969 [76]. PPARα is highly expressed in several retinal cell types (e.g., RPE, outer nuclear layer, inner nuclear layer, glial cells) and plays essential roles in ocular biology, including the regulation of VEGF expression, mitochondrial function, inflammation, apoptosis, and angiogenesis [77]. In vivo studies demonstrate that inefficient PPARα function (e.g., PPARα^-/-^ mice) results in apoptosis of retinal and pericyte cells, activation of retinal glia, and formation of retinal acellular capillaries [77,78,79]. Diabetic PPARα knockout mice exhibit increased expression of several inflammatory factors including VEGF, TNF-α, and ICAM-1, thus leading to more severe inflammation and neovascularization in the retina of diseased animals [77]. Furthermore, PPARα deficiency in diabetic mice seems to aggravate the severity of fibronectin and inflammation, as well as increasing the level of fatty acids and renal triglycerides [80,81]. On the contrary, PPARα overexpression in rats with streptozotocin(STZ)-induced diabetes reduces vascular leakage and retinal inflammation by decreasing adherent leukocytes and expression levels of VEGF, TNF-α, and ICAM-1 [77]. Recently, it has been suggested that one mechanism by which PPARα activation inhibits inflammatory responses is through the upregulation of thrombomodulin (TM) [82]. These cumulative observations suggest PPARα agonism will manifest pleiotropic downstream effects such as anti-apoptosis, anti-inflammation, and anti-oxidation—all of which would be beneficial to addressing the complex nature of prevalent retinal diseases.

**Figure 5:**
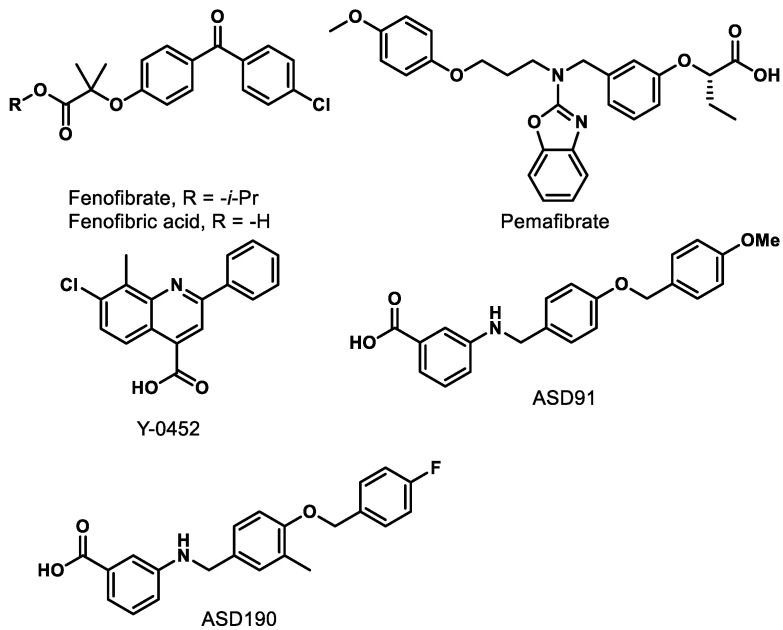
Representative synthetic PPARα agonists.

The benefits of PPARα activation have also been confirmed at the cellular level. Overexpression of PPARα in human retinal capillary endothelial cells (HRCECs) inhibits cell migration and proliferation to provide an anti-angiogenic effect [77]. In another study, protection of human retinal capillary pericytes (HRCP) was demonstrated by overexpression of PPARα, which resulted in a decrease in oxidative stress-induced apoptosis, a reduction in the production of reactive oxygen species, and a downregulation of NADPH oxidase 4 (NOX4) expression in cultured cells [78]. Mitochondrial dysfunction of HRCP was also ameliorated by the overexpression of PPARα, which reduces the production of reactive oxygen species (ROS) and thus provides protective effects [78]. PPARα overexpression inhibits the Wnt pathway and induces anti-inflammatory and anti-fibrosis effects [80]. Taken collectively, the multidimensional benefits of enhanced PPARα activity provide compelling evidence that PPARα agonism is capable of addressing the complex nature of common retinal diseases beyond what is capable with anti-VEGF strategies [78,79,80]. Two fundamental approaches can be envisioned to enhance PPARα activity: (i) genetic-induced PPARα overexpression, and (ii) ligand-induced PPARα activation. The latter option has generated excitement from academic and pharmaceutical labs, as it represents an obvious option for small-molecule drug development. Herein, we summarize recent findings for PPARα agonists (Figure 5) in the therapeutic treatment of retinal diseases.

### 9.1. Fenofibrate/Fenofibric Acid

Fenofibrate (Figure 5) is the most studied PPARα agonist for treating retinal diseases. Fibrates are amphipathic (one end is hydrophobic, and one end is hydrophilic) carboxylic acids that are employed clinically to lower plasma triglyceride levels. Fenofibrate is hydrolyzed in vivo by hepatic esterases to the active PPARα-agonizing form, fenofibric acid (Figure 5). Two preeminent studies demonstrating the beneficial effects of fenofibric acid on the progression and severity of DR are the Fenofibrate Intervention and Event Lowering in Diabetes (FIELD) study and the Action to Control Cardiovascular Risk in Diabetes (ACCORD)-Eye study [83,84].

The FIELD study evaluated the ability of long-term oral fenofibrate treatment (200 mg/day for five years) to address DR progression in a research cohort of 9795 diabetic patients [84,85]. A significant reduction in the need for the first laser treatment was observed compared to the placebo group, and the ophthalmology sub-study showed a significant reduction in the progression of retinopathy and the prevalence of macular edema in the patients with pre-existing retinopathy [84,86]. The ACCORD-Eye study explored the potential of tandem administration of fenofibrate (160 mg/day) and simvastatin (22.3 mg/day) to mitigate DR progression in a research cohort of 2856 diabetic retinopathy patients over a four-year span [83,87,88]. The combination of fenofibrate and simvastatin slowed the progression of DR, an improvement not provided by simvastatin alone. 

Since these clinical trials, the therapeutic effects of fenofibrate on DR have been found to be unrelated to its lipid-lowering activity, but rather result from its agonism and upregulation of PPARα. Given the pleiotropic role of PPARα, as described previously, it is not surprising that fenofibrate elicits protective effects against retinal neurodegeneration, pericyte dropout, inflammation, vascular leakage, and NV in OIR and type 1 diabetic models [77,78,79,89]. Furthermore, the Takahashi group reported that administration of fenofibrate prevents the upregulation of proinflammatory cytokines and monocyte chemoattractant protein-1 (MCP-1) and inhibits inflammatory cell infiltration into the injured cornea of the rats [90]. Therefore, fenofibrate treatment demonstrates beneficial effects on various pathological drivers of DR and related conditions through the activation of PPARα.

However, fenofibric acid is a weak PPARα agonist (EC_50_ = 18–30 μM) and has a poor selectivity (10–14-fold) for PPARα compared to the other two isoforms [53]. In fact, more than 100 mg/kg of fenofibrate is required to reach meaningful effects in mice studies [91]. Moreover, poor efficacy by fenofibrate to reduce cardiovascular events has resulted in fewer medical physicians prescribing it for dyslipidemia or using it off-label for treating diabetic retinopathy [92]. Although the fenofibrate results provide compelling evidence that PPARα agonism by systemically administered small molecules is a clinically validated avenue for the treatment of retinal diseases, new PPARα agonists with improved pharmacokinetic and pharmacodynamic profiles are required. 

### 9.2. Pemafibrate

**Figure 6:**
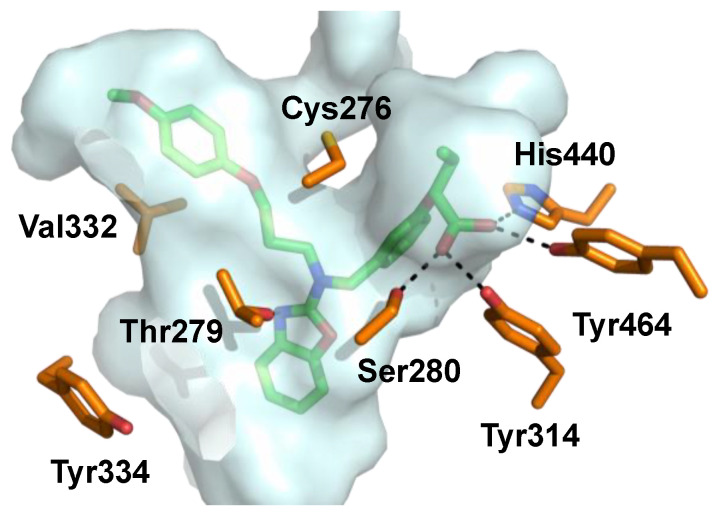
Pemafibrate (green) co-crystallized with PPARα. Binding pocket indicated with gray surface and interacting amino acids depicted in orange (PDB ID: 6L96).

Pemafibrate (K-877, Parmodia^TM^, Figure 5) is a newly approved fibrate drug developed in Japan (2017) and indicated for the treatment of atherogenic dyslipidemia. It was developed as a novel selective peroxisome proliferator-activated receptor alpha modulator (SPPARMα) and exhibits an excellent potency (EC_50_ = 1.5 nM) and a high selectivity (>2000-fold) for PPARα over other isoforms in cell-based transactivation assaysn [91,93]. Pemafibrate contains an acidic region similar to other PPARα agonists and has a unique Y-shape structure including a 2-aminobenzoxazolic ring and a phenoxyalkyl chain that provide enhanced complementarity to the topology of the PPARα binding pocket (Figure 6) [94,95,96].

Pemafibrate upregulates 11 of the top 20 genes involved in carbohydrate and lipid metabolism, such as very low density lipoprotein receptor (VLDLR), ATP binding cassette subfamily A member 1 (ABCA1), nuclear receptor co-repressor 1 and 2 (NCoR1 and NCoR2), vascular cell adhesion molecule 1 (VCAM-1), and MCP-1, but to a much greater extent than fenofibric acid [91,97]. Moreover, unique genes that are involved in regulation of the innate immune system and inflammation, such as mannose-binding lectin 2 (MBL2), glutamyl aminopeptidase (ENPEP), and fibroblast growth factor 21 (FGF21), are induced following pemafibrate administration [98]. 

Similar to fenofibrate, pemafibrate exhibits beneficial effects on lipid metabolism and inflammation through the activation of PPARα [99,100]. Administration of pemafibrate in LDL receptor knockout mice results in a reduction in plasma triglycerides and total cholesterol, and a concomitant increase in high-density lipoprotein cholesterol (HDL-C) as a result of PPARα-related gene regulation [99]. In human apolipoprotein E2 knock-in mice, pemafibrate reduces biomarkers for inflammation and macrophages, such as MCP1, VCAM-1, and interleukin 6 (IL6) [100]. Moreover, the low therapeutic dose of pemafibrate (0.2–0.4 mg/day) is unlikely to induce peroxisome proliferation or liver toxicity in clinical settings [100]. In fact, pemafibrate showed only a 25% increase in liver weight compared to the 44% increase of fenofibrate in rats, suggesting chemotype-dependent side effects for fenofibrate and an improved side effect profile for pemafibrate [94].

Inspired by the fenofibrate FIELD and ACCORD studies, pemafibrate was scheduled to undergo a phase III clinical trial, called Pemafibrate to Reduce Cardiovascular OutcoMes by Reducing Triglycerides IN patiENts with diabeTes (PROMINENT), which was scheduled for March 2017 in the United States and Europe [101]. The PROMINENT study was not only expected to investigate effects of pemafibrate on the residual cardiovascular risk remaining after treatment but also the ability of pemafibrate to reduce DR in diabetic patients through an ancillary study [102]. Unfortunately, the initial recruiting period failed to reach the required enrollment and the trial has been postponed. Recent studies show that pemafibrate, but not fenofibrate, inhibits ischemia-induced retinal angiogenesis in an animal model [103]. Surprisingly it has been suggested that systemically administered pemafibrate elicits this effect indirectly through the agonism of liver PPARα rather than from directly stimulating PPARα in the retina [103]. Although more studies are needed, pemafibrate represents a promising therapeutic lead to treat inflammatory and neovascular retinal diseases.

### 9.3. Y-0452

Recently, a new PPARα agonist, 7-chloro-8-methyl-2-phenylquinoline-4-carboxylic acid (Y-0452, Figure 5), was reported by the Ma Lab at the University of Oklahoma Health Science Center [104]. Y-0452 was identified from a virtual screen as a chemically distinct chemotype predicted to exhibit PPARα agonism [104]. Experimentally, the compound exhibits anti-apoptosis and neuroprotective effects in R28 (a cell line derived from photoreceptor precursors) and an anti-angiogenic effect in HRCECs [104]. Additionally, the compound significantly reduces retinal inflammation and apoptosis without signs of toxicity in the retinas of mice and diabetic rats [104]. Y-0452 exhibits efficacy in DR animal models after systemic (i.p.) administration, providing a new lead for the development of novel PPARα agonists [104]. Although Y-0452 represents a novel PPARα agonistic chemotype, it exhibits only weak on-target activity in biochemical PPARα assays (EC_50_ ≈ 25–50 µM), and manifests a low level of agonism compared to known PPARα agonists [104]. Additionally, the highly functionalized quinoline core of Y-0452 presents significant synthetic challenges regarding comprehensive structure–activity relationship (SAR) studies. These aspects inspired us to investigate the SAR of Y-0452 through molecular simplification with a goal of enhancing synthetic tractability, target engagement, selectivity, and level of PPARα agonism. Towards this initiative, we utilized in silico approaches to design a series of derivatives, which were then synthesized and evaluated for PPARα agonism. 

### 9.4. A91 and A190

From in silico studies surrounding a Y-0452•PPARα binding model, we hypothesized that carboxylic acid transposition and deconstruction of the Y-0452 quinoline system would enhance ligand–protein interactions and better complement the nature of the binding pocket [105]. The initial interrogation of this hypothesis produced a novel class of 4-benzyloxy-benzylamino PPARα agonists, from which A91 (Figure 5) was identified as exhibiting an EC_50_ = ~4 µM and manifesting > 20-fold selectivity for PPARα over the PPARγ and PPARδ isoforms [105]. Further evaluation confirmed the PPARα activation of A91 including PPARα upregulation, induction of target genes (e.g., *Acadm*, *Cpt1a*, *Fabp3*, *Slc25a20*) in 661W cells, and inhibition of cell migration [105]. 

With the confirmation of PPARα activation by A91, we recently evaluated its in vivo activity in a well-established STZ-induced rat model of retinal vascular leakage [106]. Daily administration of A91 (25 mg/kg/day, i.p.) was shown to reduce retinal vascular leakage to non-diabetic levels at relatively the same dose as FenoFA [106]. Of interesting note, A91 appears to lack signs of hepatomegaly, a dose-limiting toxicity often observed with fenofibrate [107]. Furthermore, A91 proves to be stable in both human and rat microsomes (t_1/2_ > 60 min), exhibits no evidence of irreversible inhibition of major drug-metabolizing CYP450 enzymes (1A, 2C9, 2C19, 2D6, 3A), and lastly lacks hERG inhibition [106]. The results demonstrate that A91 (i) exhibits in vivo efficacy in a relevant DR model following systemic administration, (ii) is bioavailable, (iii) survives first-pass metabolism and clearance mechanisms well enough to maintain efficacy, and (iv) demonstrates a relatively safe profile (no observable toxicity after daily treatment for a month) [106]. Recently, we advanced the SAR on this chemotype to provide analog A190 (Figure 5), which capitalizes on a methyl effect rationally designed through structure-based approaches. This compound exhibits an EC_50_ of ~40 nM in the cell-based luciferase assay (a ~100-fold improvement from A91), exhibits > 2700-fold selectivity over other PPAR isoforms, and maintains a promising initial PK profile [106]. Additional congeners of this series that exhibit improved cellular potencies have been developed and put this non-fibrate scaffold on par with pemafibrate in terms of potency and selectivity.

## 10. Strategic Promiscuity

### 10.1. Dual PPAR Regulation

Various PPAR dual agonists, especially PPARα/γ dual agonists, and PPAR pan(α/δ/γ) agonists have emerged in recent years, including lobeglitazone sulfate (approved in Korea), aleglitazar (Roche), ragaglitazar (Novo Nordisk), imiglitazar (Takeda), peliglitazar (Bristol-Myers Squibb), and farglitazar (GlaxoSmithKline) [108]. Recently, it has been reported that the PPARα/γ dual agonist saroglitazar, developed by Zydus cadila and approved in India, is patented for treating retinal diseases caused by inflammation, macular degeneration, and neovascularization [109]. However, the development of dual PPAR agonists has not yet achieved the anticipated success in the United States, due to the side effects such as increased cardiovascular risk (muraglitazear), [110] carcinogenicity (ragaglitazar and MK-767), [111] liver toxicity (imiglitazar), [112] and renal injury (tesaglitazar) [112].

### 10.2. PPAR and FABP

**Figure 7:**
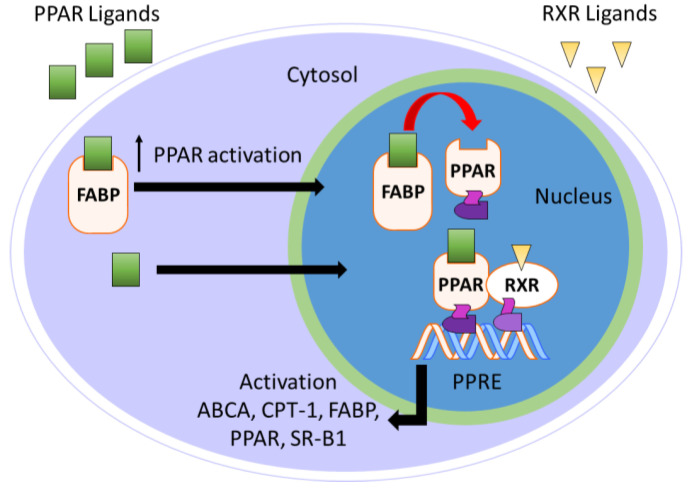
Overview of FABP-mediated PPAR activation.

Fatty acid-binding proteins (FABPs) are a group of 14–15 kDa intracellular cytosolic lipid-binding proteins. Research on FABPs and their biological importance has garnered interest from researchers in both academic and pharmaceutical settings [113,114,115,116]. FABPs facilitate the transport of free fatty acids to many specific compartments in the cell for storage, signaling, and/or metabolism (Figure 7) [116]. Most relevant to the topic of this perspective, FABPs transport hydrophobic ligands to PPARs, thereby enhancing transcriptional regulation [117]. Without FABP shuttling, the hydrophobic nature of PPAR ligands would preclude interactions with PPARs.

FABPs 1–9 have attracted the most interest from researchers to date. Research shows that FABPs coexist in tissue or cells, so the numeric nomenclature is preferred over the more historical tissue-related names: FABP1 (liver/L-), FABP2 (intestinal/I-), FABP3 (heart/H-), FABP4 (adipocyte/A-), FABP5 (epidermal/E-), FABP6 (ileal/Il-), FABP7 (brain/B-), FABP8 (myelin/M-), and FABP9 (testis/T-) [115,116]. Recently, a new FABP isoform, FABP12, was found mainly in the retina including in the ganglion cells and the inner nuclear layer of adult rats [118]. Other FABPs, such as FABP7, have also been found in the retina and play an important role in maintenance of retinal vasculature [119]. This suggests that FABPs are likely to play an important role in the PPAR ligand shuttling in the retina, and thus are presumably involved in the pathology of retinal diseases.

**Figure 8:**
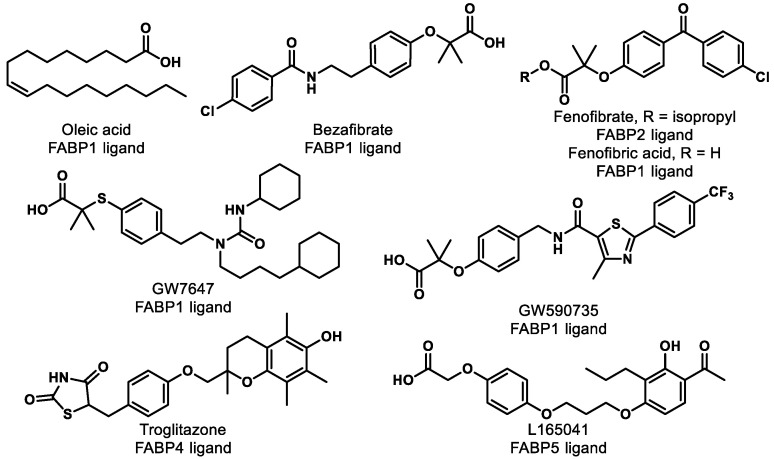
Compounds listed in Table 2.

Research shows that FABPs promote the uptake and transportation of long-chain fatty acids or synthetic ligands to PPARs, thereby enhancing the ability of these ligands to interact with PPARs [120]. Interestingly, studies have suggested that FABPs are fairly selective for ligands and specific PPARs (Table 2) [120,121]. For example, multiple groups have demonstrated that FABP1 interacts with PPARα and PPARγ but not with PPARδ [120,122,123]. More specifically, FABP1 and FABP2 enhance the transcriptional activation of PPARα by delivery of oleic acid and hypolipidemic drugs, such as fenofibric acid and GW7647 (Figure 8) [124]. The activations of PPARγ and PPARδ are increased by FABP4 and FABP5, respectively [125]. Moreover, genes expressing FABPs are transcribed by the activation of PPARs [126,127]. Thus, the hypothesis arises that ligand affinity should be optimized for both targets (i.e., FABP and PPAR) if one wants to optimize PPAR agonism. For PPARα, for example, one might focus on optimizing affinity for FABP1 and PPARα simultaneously to achieve the optimal efficiency of activation. This strategy has yet to be employed in the literature, as far as we can tell, and represents an exciting unexplored avenue for future studies.

#### 10.3. PPAR and RXR

**Figure 9:**
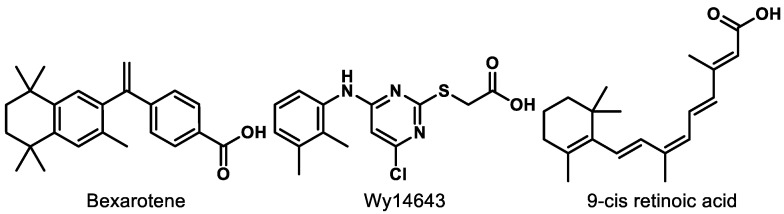
Compounds mentioned for dual PPAR–RXR interaction.

RXRs are well known as the heterodimerization partner of many nuclear receptors (NRs) such as constitutive androstane receptor (CAR), farnesoid X receptor (FXR), liver X receptor (LXR), and PPAR [128,129]. There are three subtypes of RXRs including RXRα, RXRβ, and RXRγ. Activation of RXRs is known to elicit beneficial effects for inflammation, [130] central nervous system (CNS) remyelination, [131] multiple sclerosis (MS), [132,133] Alzheimer’s disease (AD), [134] and cancer [135]. However, RXR agonism as a therapeutic approach is still at a relatively preliminary stage. The only market-approved RXR agonist, bexarotene (Figure 9), has faced safety issues including skin dryness, hypothyroidism, and hypertriglyceridemia [136,137,138]. Gemfibrozil, a fibrate drug that is used to improve cholesterol and triglyceride levels, was originally intended to reduce the side effects of bexarotene but resulted in a worsened condition due to an increase in bexarotene in plasma [136]. 

Dual RXR and PPAR (especially PPARα) agonism, however, might provide new avenues for PPAR-mediated treatments. As mentioned above, RXR is the heterodimeric partner of many NRs and increasing evidence indicates that RXR impacts the response of its NR partners. The interactions of RXR and its heterodimeric partners have been classified into three groups: permissive, non-permissive, and conditionally permissive [139]. A permissive NR in the context of an RXR heterodimer can be partially activated with an RXR or NR agonist, but both ligands are required for full agonism [129]. In non-permissive NR/RXR heterodimers, no response is observed in the presence of only the RXR ligand, but full levels of agonism result from the presence of the NR receptor. In conditionally permissive systems, synergy or increased activity with the NR and RXR agonists are observed, but no activity results from the RXR agonist alone. 

In the context of PPARα, the system is permissive and thus both molecules are required for full levels of agonism. This may explain the inconsistencies in behavior of known PPARα agonists in LBD-Gal4 versus full-length PPAR/RXR engineered luciferase systems [140]. The generally accepted theory is that, in permissive NR/RXR systems, conformational cross-talk between receptors in the presence or absence of corresponding ligands is what drives the cooperation [139]. As such, it is expected that activation of PPAR/RXR heterodimers by different ligands could lead to significant alterations in the dimer conformation and thus differential profiles of gene expression [139,141]. The identification or design of RXR/PPARα dual binders, for example, would not only provide chemical probes to aid in the understanding of permissive regulation, but also give rise to drug leads with more predictable pharmacodynamic profiles. It is tempting to speculate that dual binders that give rise to alternative receptor conformations may provide tunable small-molecule gene regulation systems and new leads for retinal diseases. 

Wy14643 (Figure 9), a known PPARα agonist, was found to be more potent for RXR than PPARs, which could be the reason why Wy14643 manifests unique biological activities compared to other PPARα agonists, such as clofibrate [142]. The dual RXR/PPARα agonism did not trigger the sources of side effects in bexarotene therapy from the in vitro and in vivo studies [142]. Moreover, research shows that activation of RXR improves the transcription by PPARs in a permissive way [129]. PPAR-mediated gene expression was increased more than 3-fold by the addition of RXR when PPAR was activated by 9-*cis* retinoic acid (Figure 9) [143]. 

## 11. Perspective/Conclusions

Retinal diseases, such as AMD, DR, and ROP, have become widespread serious medical conditions. However, the current treatments are still insufficient, lack efficacy in certain stages of the diseases (as in the case for AMD), or exhibit detrimental side effects (such as for DR or ROP). The development of new treatments is necessary. The pathology of vascular-related retinal diseases spans an extensive web of molecular pathways and networks, such as lipid accumulation- or oxidative stress-induced inflammation, upregulated angiogenic factors (e.g., VEGF) causing aberrant angiogenesis, and NV, leading to retinal detachments [10]. Diverse higher-order physiological activities such as Bruch’s membrane homeostasis, protein and lipid turnover, energy metabolism, and complement regulation are also involved in the disease etiology. Further, the complex anatomical microenvironments in the retina are a critical consideration when addressing retinal diseases (e.g., blood–retina barrier). 

PPAR modulation has been investigated rather extensively over recent decades as a treatment strategy for a variety of diseases. Thus, while the interrogation of this strategy in the context of retinal physiology is relatively nascent, a rich breadth of literature provides clues as to what challenges lie ahead as this field continues to advance. For example, (i) demonstrating long-term safety, (ii) determining the optimal route of administration (i.e., systemic (oral or i.v.), ocular (topical, intraocular)) and defining acceptable absorption/distribution profiles, (iii) determining if sufficient efficacy exists to provide stand-alone capability; (iv) determining the optimal isoform (or combination) to modulate for efficacy; (v) demonstrating a lack of off-target effects; and (vi) overcoming a rather unsuccessful history of PPAR modulator development. It is encouraging to note, however, that although several PPAR modulators have been abandoned for previous indications, the reasons for discontinuations have been due to chemotype-specific issues or reasons unrelated to PPAR modulation. Identifying and developing organ compatible chemotypes with improved ADMET profiles represent a tangible and realistic endeavor. In addition to the aforementioned challenges, critical/interesting questions persist in the field that deserve attention, including but not limited to: What level of modulation is optimal, especially in the context of agonism (i.e., full or partial)? Can predictive biomarkers be identified for vascular-related retinal diseases that respond to pharmacological interventions? In developing ligands, is potency or efficacy more important? What are the molecular mechanisms behind PPAR involvement in vascular-related retinal diseases? Can PPAR modulation provide prophylactic protection? Answers to these questions will inevitably open new avenues of inquiry and drive medicinal chemistry campaigns.

New approaches for DR, AMD, and ROP should be able to address the complex interplay of pathogenic factors, be mechanistically differentiated from current strategies, provide superior and/or synergistic effects on current treatments, and be capable of being employed as prophylactics for high-risk populations. New diagnostic tools (e.g., biomarkers) that can identify preclinical risk factors or subtypes of retinal disease to prevent and/or predict the progression at early stages are also crucial for the development of new approaches. Activation of PPAR, especially PPARα, which has demonstrated efficacy in the clinic, with small molecules is a promising strategy to treat various retinal diseases. Development of systemically available options for treating retinal diseases will provide patients a safe, readily available, affordable treatment that will undoubtedly enhance patient quality of life.

## Figures and Tables

**Table 1:** Current anti-VEGF agents.

Drug (Brand Name)	Sponsor	FDA-Approved Indications
Pegaptanib sodium (Macugen)	Eyetech/Pfizer	Macular degeneration
Ranibizumab (Lucentis)	Genentech/Roche	Macular degeneration, macular edema, myopic choroidal neovascularization, diabetic macular edema, and DR
Aflibercept (Eylea)	Regeneron	Macular degeneration, macular edema, and DR
Bevacizumab (Avastin)	Genentech	Colorectal cancer, non-small cell lung cancer, glioblastoma multiforme, renal cell carcinoma, cervical cancer, ovarian cancer, fallopian tube cancer, and peritoneal cancer

**Table 2:** PPAR ligands interact with FABPs.

Ligand	PPARs	FABPs	Note	Refs
**Oleic acid**	PPARα	FABP1	A linear correlation is shown between transactivation and FABP1 concentration. Binding affinity of h-FABP1 is 0.15 μM. Binding of PPARα is 0.21 μM.	[120,122,123]
**Bezafibrate**	PPARα	FABP1	Linear correlation is shown between transactivation and FABP1 concentration.	[123]
**Fenofibrate**	PPARα	FABP2	2-fold increase	[124]
**Fenofibric acid**	PPARα	FABP1	Binding affinity of h-FABP1 is 1.0 μM. Binding affinity of PPARα is 10 μM.	[123,124]
**GW7647**	PPARα	FABP1	Binding affinity of h-FABP1 is 0.32 μM. Binding affinity of PPARα is 0.035 μM.	[122,123]
**GW590735**	PPARα	FABP1	Binding affinity of h-FABP1 is 20 μM. Binding affinity of PPARα is 0.06 μM	[123]
**Troglitazone**	PPARγ	FABP4	Linear correlation between transactivation and FABP4 concentration. 1.5-fold increase was improved with 0.3 µg of FABP4. Binding affinity of FABP4 is 47.3 nM. Binding affinity of PPARγ is 50.7 nM.	[125]
**L165041**	PPARβ	FABP5	Liner correlation between transactivation and FABP5 concentration. 3-fold increase was improved with 0.3 µg of FABP5. Binding affinity of FABP5 is 45.9 nM. Binding affinity of PPARβ is 33.1 nM.	[123,125]
